# Supplementary material for: Ropivacaine inhibits the malignant behavior of lung cancer cells by regulating retinoblastoma-binding protein 4
Source: PeerJ. 2023 Nov 27;11:e16471. doi: 10.7717/peerj.16471 (PMC10688306; doi:10.7717/peerj.16471)
Supplement: Supplemental Information 1 [file peerj-11-16471-s001.docx]

**Supplementary Table 1 DEPs between ropivacaine-treated groups and control groups in A549 cells**

| number | accession | gene symbol | number | accession | gene symbol |
| --- | --- | --- | --- | --- | --- |
| 1 | P08779 | KRT16 | 2 | P02538 | KRT6A |
| 3 | Q9BZG1 | RAB34 | 4 | P48307 | TFPI2 |
| 5 | O75521 | ECI2 | 6 | Q58FG1 | HSP90AA4P |
| 7 | P63279 | UBE2I | 8 | O75841 | UPK1B |
| 9 | Q9UKL0 | RCOR1 | 10 | P11441 | UBL4A |
| 11 | P41223 | BUD31 | 12 | **Q09028** | **RBBP4** |
| 13 | P60903 | S100A10 | 14 | Q9NX70 | MED29 |
| 15 | O00559 | EBAG9 | 16 | Q9BPX5 | ARPC5L |
| 17 | P61088 | UBE2N | 18 | P10109 | FDX1 |
| 19 | P57105 | SYNJ2BP | 20 | Q7Z4V5 | HDGFL2 |
| 21 | P40222 | TXLNA | 22 | Q13310 | PABPC4 |
| 23 | P69905 | HBA1 | 24 | P06132 | UROD |
| 25 | P36954 | POLR2I | 26 | Q6NZ67 | MZT2B |
| 27 | P20290 | BTF3 | 28 | P98194 | ATP2C1 |
| 29 | Q9UKJ3 | GPATCH8 | 30 | Q14444 | CAPRIN1 |
| 31 | P40855 | PEX19 | 32 | Q9NX24 | NHP2 |
| 33 | Q15404 | RSU1 | 34 | P62273 | RPS29 |
| 35 | Q15388 | TOMM20 | 36 | P50151 | GNG10 |
| 37 | Q13123 | IK | 38 | P55061 | TMBIM6 |
| 39 | Q96ST2 | IWS1 | 40 | Q9BRK5 | SDF4 |
| 41 | Q8WXX5 | DNAJC9 | 42 | Q9C0B1 | FTO |
| 43 | O60232 | ZNRD2 | 44 | P52292 | KPNA2 |
| 45 | O15347 | HMGB3 | 46 | Q13283 | G3BP1 |
| 47 | P54105 | CLNS1A | 48 | Q16763 | UBE2S |
| 49 | Q6P2E9 | EDC4 | 50 | Q9GZM8 | NDEL1 |
| 51 | P06454 | PTMA | 52 | O95626 | ANP32D |
| 53 | P49321 | NASP | 54 | Q9H0R4 | HDHD2 |
| 55 | P16989 | YBX3 | 56 | P62256 | UBE2H |
| 57 | P30519 | HMOX2 | 58 | P0C0S5 | H2AZ1 |
| 59 | Q15027 | ACAP1 | 60 | Q99417 | MYCBP |
| 61 | Q5EBL4 | RILPL1 | 62 | Q96HQ2 | CDKN2AIPNL |
| 63 | Q53H82 | LACTB2 | 64 | Q9H201 | EPN3 |
| 65 | P02533 | KRT14 | 66 | Q9H446 | RWDD1 |
| 67 | Q5T6F2 | UBAP2 | 68 | Q9P0U1 | TOMM7 |
| 69 | P12004 | PCNA | 70 | P62633 | CNBP |
| 71 | O75718 | CRTAP | 72 | P06899 | H2BC11 |
| 73 | Q8TED1 | GPX8 | 74 | Q12999 | TSPAN31 |
| 75 | P60468 | SEC61B | 76 | Q92896 | GLG1 |
| 77 | Q8IYB3 | SRRM1 | 78 | P46013 | MKI67 |
| 79 | P41208 | CETN2 | 80 | P62310 | LSM3 |
| 81 | Q9UK76 | JPT1 | 82 | O00483 | NDUFA4 |
| 83 | Q99729 | HNRNPAB | 84 | Q99614 | TTC1 |
| 85 | P67809 | YBX1 | 86 | Q99439 | CNN2 |
| 87 | Q9BV40 | VAMP8 | 88 | Q13442 | PDAP1 |
| 89 | Q99653 | CHP1 | 90 | Q01995 | TAGLN |
| 91 | Q9BVG4 | PBDC1 | 92 | G2XKQ0 | SUMO1P1 |
| 93 | Q14157 | UBAP2L | 94 | O43290 | SART1 |
| 95 | Q9Y3D0 | CIAO2B | 96 | O15231 | ZNF185 |
| 97 | Q9UJ72 | ANXA10 | 98 | Q13263 | TRIM28 |
| 99 | P05387 | RPLP2 | 100 | P13693 | TPT1 |
| 101 | P43487 | RANBP1 | 102 | P56385 | ATP5ME |
| 103 | P26583 | HMGB2 | 104 | Q92688 | ANP32B |
| 105 | Q09161 | NCBP1 | 106 | P11717 | IGF2R |
| 107 | Q15637 | SF1 | 108 | Q9Y224 | RTRAF |
| 109 | P00167 | CYB5A | 110 | Q96ST3 | SIN3A |
| 111 | Q9Y3C1 | NOP16 | 112 | O95232 | LUC7L3 |
| 113 | Q08752 | PPID | 114 | P17275 | JUNB |
| 115 | P83916 | CBX1 | 116 | Q12874 | SF3A3 |
| 117 | O75934 | BCAS2 | 118 | P61289 | PSME3 |
| 119 | Q9Y383 | LUC7L2 | 120 | Q13813 | SPTAN1 |
| 121 | Q96RS6 | NUDCD1 | 122 | Q9Y5S9 | RBM8A |
| 123 | P84090 | ERH | 124 | P24534 | EEF1B2 |
| 125 | O15355 | PPM1G | 126 | P61457 | PCBD1 |
| 127 | P48634 | PRRC2A | 128 | P09496 | CLTA |
| 129 | P62851 | RPS25 | 130 | Q9BVL2 | NUP58 |
| 131 | P35269 | GTF2F1 | 132 | Q92820 | GGH |
| 133 | P22392 | NME2 | 134 | P50213 | IDH3A |
| 135 | O14776 | TCERG1 | 136 | P06753 | TPM3 |
| 137 | Q9NYL4 | FKBP11 | 138 | Q15287 | RNPS1 |
| 139 | O75607 | NPM3 | 140 | Q8N5K1 | CISD2 |
| 141 | Q9UHV9 | PFDN2 | 142 | Q8ND56 | LSM14A |
| 143 | P62917 | RPL8 | 144 | P53992 | SEC24C |
| 145 | P39656 | DDOST | 146 | P30044 | PRDX5 |
| 147 | Q05193 | DNM1 | 148 | P99999 | CYCS |
| 149 | Q8NF37 | LPCAT1 | 150 | Q71UM5 | RPS27L |
| 151 | Q14108 | SCARB2 | 152 | Q9BSJ2 | TUBGCP2 |
| 153 | Q9NX58 | LYAR | 154 | P15121 | AKR1B1 |
| 155 | P60900 | PSMA6 | 156 | Q9UHB9 | SRP68 |
| 157 | P11216 | PYGB | 158 | Q9NQR4 | NIT2 |
| 159 | Q16850 | CYP51A1 | 160 | A0FGR8 | ESYT2 |
| 161 | P53004 | BLVRA | 162 | Q9Y512 | SAMM50 |
| 163 | P40939 | HADHA | 164 | Q53GQ0 | HSD17B12 |
| 165 | Q16881 | TXNRD1 | 166 | P21399 | ACO1 |
| 167 | P24752 | ACAT1 | 168 | P16435 | POR |
| 169 | P40926 | MDH2 | 170 | Q05639 | EEF1A2 |
| 171 | O75251 | NDUFS7 | 172 | P56134 | ATP5MF |
| 173 | P06737 | PYGL | 174 | P78527 | PRKDC |
| 175 | Q9UJS0 | SLC25A13 | 176 | P31327 | CPS1 |
| 177 | O15143 | ARPC1B | 178 | P16930 | FAH |
| 179 | Q9P035 | HACD3 | 180 | Q96S97 | MYADM |
| 181 | Q9Y2A7 | NCKAP1 | 182 | P09525 | ANXA4 |
| 183 | Q7Z478 | DHX29 | 184 | Q9NVK5 | FGFR1OP2 |
| 185 | P35908 | KRT2 | 186 | P14618 | PKM |
| 187 | Q92747 | ARPC1A | 188 | P09972 | ALDOC |
| 189 | Q15067 | ACOX1 | 190 | P56556 | NDUFA6 |
| 191 | Q14697 | GANAB | 192 | P35611 | ADD1 |
| 193 | Q9Y277 | VDAC3 | 194 | P12236 | SLC25A6 |
| 195 | Q5SSJ5 | HP1BP3 | 196 | O60218 | AKR1B10 |
| 197 | P42765 | ACAA2 | 198 | Q00325 | SLC25A3 |
| 199 | Q99685 | MGLL | 200 | Q13151 | HNRNPA0 |
| 201 | Q9NR45 | NANS | 202 | P22695 | UQCRC2 |
| 203 | Q15738 | NSDHL | 204 | Q16831 | UPP1 |
| 205 | O00425 | IGF2BP3 | 206 | Q6UW68 | TMEM205 |
| 207 | P21281 | ATP6V1B2 | 208 | O43813 | LANCL1 |
| 209 | P10253 | GAA | 210 | P83881 | RPL36A |
| 211 | Q5SWX8 | ODR4 | 212 | Q9BS40 | LXN |
| 213 | Q8N3U4 | STAG2 | 214 | P15428 | HPGD |
| 215 | P00367 | GLUD1 | 216 | O75396 | SEC22B |
| 217 | P08559 | PDHA1 | 218 | O15533 | TAPBP |
| 219 | Q6NUK1 | SLC25A24 | 220 | Q08380 | LGALS3BP |
| 221 | Q14573 | ITPR3 | 222 | Q9NRW7 | VPS45 |
| 223 | Q7Z2W4 | ZC3HAV1 | 224 | Q99536 | VAT1 |
| 225 | Q15436 | SEC23A | 226 | P02787 | TF |
| 227 | Q96EP5 | DAZAP1 | 228 | Q7Z7K6 | CENPV |
| 229 | Q9Y6A9 | SPCS1 | 230 | Q00535 | CDK5 |
| 231 | P25685 | DNAJB1 | 232 | P04040 | CAT |
| 233 | O14841 | OPLAH | 234 | P51153 | RAB13 |
| 235 | O60701 | UGDH | 236 | P05091 | ALDH2 |
| 237 | Q08257 | CRYZ | 238 | Q96HC4 | PDLIM5 |
| 239 | P25787 | PSMA2 | 240 | P49902 | NT5C2 |
| 241 | P42126 | ECI1 | 242 | Q8NCA5 | FAM98A |
| 243 | Q6UXN9 | WDR82 | 244 | P49327 | FASN |
| 245 | O00159 | MYO1C | 246 | Q6IBS0 | TWF2 |
| 247 | Q01628 | IFITM3 | 248 | O96013 | PAK4 |
| 249 | Q16851 | UGP2 | 250 | Q14534 | SQLE |
| 251 | P49753 | ACOT2 | 252 | Q9Y281 | CFL2 |
| 253 | Q16795 | NDUFA9 | 254 | P00846 | MT-ATP6 |
| 255 | P14324 | FDPS | 256 | P26640 | VARS1 |
| 257 | Q9UBX3 | SLC25A10 | 258 | Q6PIU2 | NCEH1 |
| 259 | P13804 | ETFA | 260 | Q9H3K6 | BOLA2 |
| 261 | Q53S08 | RAB6D | 262 | Q15125 | EBP |
| 263 | P53007 | SLC25A1 | 264 | Q13045 | FLII |
| 265 | O00743 | PPP6C | 266 | Q9Y4P3 | TBL2 |
| 267 | Q9NZ01 | TECR | 268 | P07099 | EPHX1 |
| 269 | P20339 | RAB5A | 270 | Q96SQ9 | CYP2S1 |
| 271 | Q9BPW8 | NIPSNAP1 | 272 | P31930 | UQCRC1 |
| 273 | Q92879 | CELF1 | 274 | Q03001 | DST |
| 275 | P35237 | SERPINB6 | 276 | Q12929 | EPS8 |
| 277 | Q86YZ3 | HRNR | 278 | O60256 | PRPSAP2 |
| 279 | Q8NBX0 | SCCPDH | 280 | Q13423 | NNT |
| 281 | Q9BWD1 | ACAT2 | 282 | Q96N66 | MBOAT7 |
| 283 | Q92797 | SYMPK | 284 | P62140 | PPP1CB |
| 285 | Q9Y394 | DHRS7 | 286 | Q15629 | TRAM1 |
| 287 | O75964 | ATP5MG | 288 | Q8NBQ5 | HSD17B11 |
| 289 | P11166 | SLC2A1 | 290 | P13645 | KRT10 |
| 291 | Q8TC12 | RDH11 | 292 | Q07352 | ZFP36L1 |
| 293 | O96008 | TOMM40 | 294 | Q16762 | TST |
| 295 | P12955 | PEPD | 296 | O95340 | PAPSS2 |
| 297 | Q9GZP9 | DERL2 | 298 | P17252 | PRKCA |
| 299 | O00560 | SDCBP | 300 | Q04446 | GBE1 |
| 301 | Q9BRX8 | PRXL2A | 302 | P37268 | FDFT1 |
| 303 | P35354 | PTGS2 | 304 | P04264 | KRT1 |
| 305 | Q9Y2J8 | PADI2 | 306 | O15126 | SCAMP1 |
| 307 | P48637 | GSS | 308 | O43237 | DYNC1LI2 |
| 309 | Q9UI12 | ATP6V1H | 310 | Q96HS1 | PGAM5 |
| 311 | Q9NW13 | RBM28 | 312 | Q969H8 | MYDGF |
| 313 | P55084 | HADHB | 314 | P30740 | SERPINB1 |
| 315 | P12931 | SRC | 316 | O75746 | SLC25A12 |
| 317 | Q14554 | PDIA5 | 318 | P00387 | CYB5R3 |
| 319 | P35527 | KRT9 | 320 | P53801 | PTTG1IP |
| 321 | Q86SF2 | GALNT7 | 322 | Q92504 | SLC39A7 |
| 323 | P36873 | PPP1CC | 324 | Q96G23 | CERS2 |
| 325 | Q99598 | TSNAX | 326 | O94952 | FBXO21 |
| 327 | Q06136 | KDSR |  |  |  |
