# Supplementary figures and images for: Ropivacaine inhibits the malignant behavior of lung cancer cells by regulating retinoblastoma-binding protein 4
Source: PeerJ. 2023 Nov 27;11:e16471. doi: 10.7717/peerj.16471 (PMC10688306; doi:10.7717/peerj.16471)

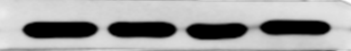

Supplement: Supplemental Information 2 [file peerj-11-16471-s002.zip › original data/figure 4a western GAPDH.png]

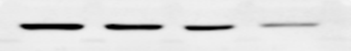

Supplement: Supplemental Information 2 [file peerj-11-16471-s002.zip › original data/figure 4a wstern RBBP4.png]

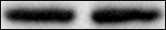

Supplement: Supplemental Information 3 [file peerj-11-16471-s003.zip › supplementary figure 2/A549 Overexpression GAPDH.tif]

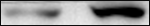

Supplement: Supplemental Information 3 [file peerj-11-16471-s003.zip › supplementary figure 2/A549 Overexpression.tif]

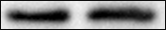

Supplement: Supplemental Information 3 [file peerj-11-16471-s003.zip › supplementary figure 2/H1299 overexpression GAPDH.tif]

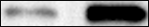

Supplement: Supplemental Information 3 [file peerj-11-16471-s003.zip › supplementary figure 2/H1299 overexpression.tif]

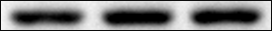

Supplement: Supplemental Information 3 [file peerj-11-16471-s003.zip › supplentary figure 1/A549 GAPDH weatern.tif]

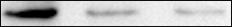

Supplement: Supplemental Information 3 [file peerj-11-16471-s003.zip › supplentary figure 1/A549 RBBP4 western.tif]

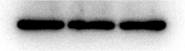

Supplement: Supplemental Information 3 [file peerj-11-16471-s003.zip › supplentary figure 1/H1299 GAPDH wetern.png]

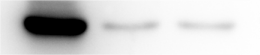

Supplement: Supplemental Information 3 [file peerj-11-16471-s003.zip › supplentary figure 1/H1299 RBBP4 wetswen.png]

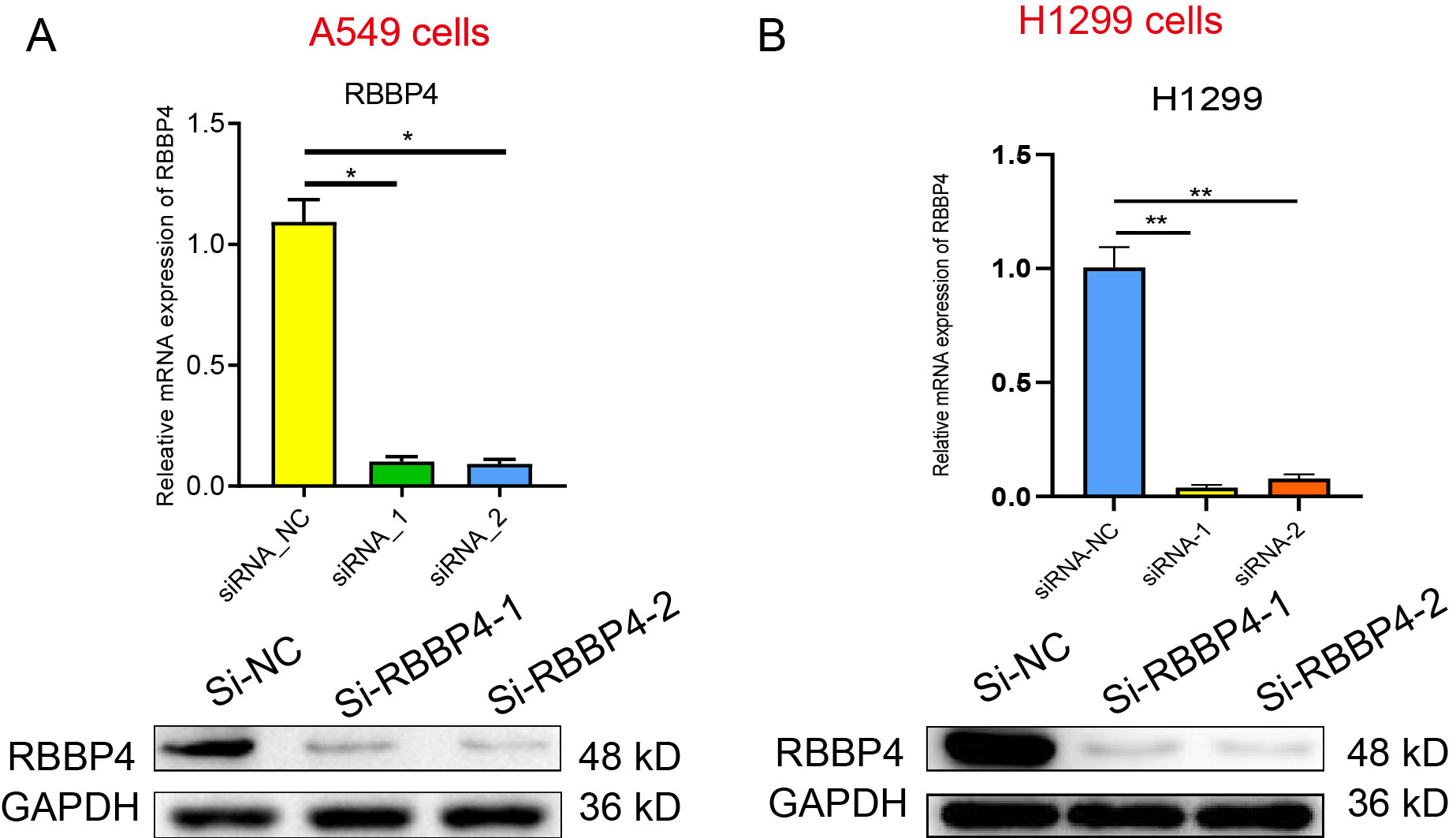

Supplement: Supplemental Information 4 [file peerj-11-16471-s004.jpg]
