# Supplementary material for: Ropivacaine inhibits the malignant behavior of lung cancer cells by regulating retinoblastoma-binding protein 4
Source: PeerJ. 2023 Nov 27;11:e16471. doi: 10.7717/peerj.16471 (PMC10688306; doi:10.7717/peerj.16471)
Supplement: Supplemental Information 5 [file peerj-11-16471-s005.pdf]

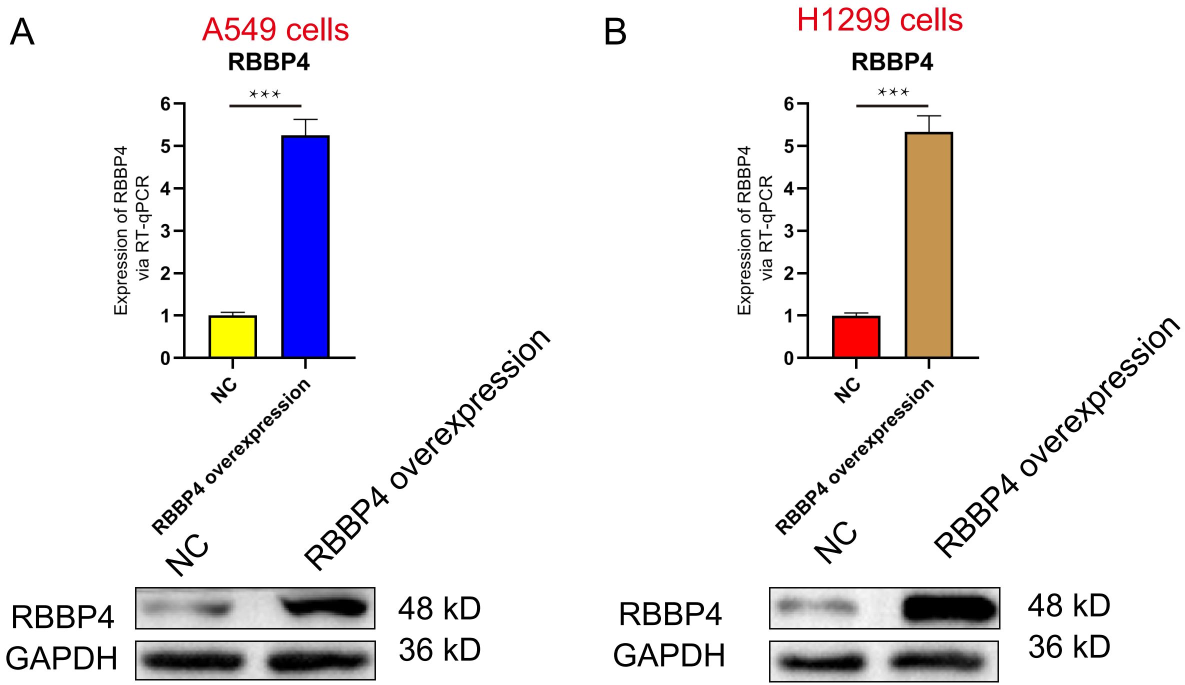
A
A549 cells
RBBP4
***
Expression of RBBP4 via RT-qPCR
0
1
2
3
4
5
6
NC
RBBP4 overexpression
NC
RBBP4 overexpression
RBBP4
GAPDH
48 kD
36 kD
B
H1299 cells
RBBP4
***
Expression of RBBP4 via RT-qPCR
0
1
2
3
4
5
6
NC
RBBP4 overexpression
NC
RBBP4 overexpression
RBBP4
GAPDH
48 kD
36 kD
